#  Small-Area Analysis of Incidence and Localisation of Vulvar Cancer

**DOI:** 10.1155/2010/512032

**Published:** 2010-06-22

**Authors:** Klaus H. Baumann, Olga Müller, Helke B. Naujok, Ellen Mann, Peter Barth, Uwe Wagner

**Affiliations:** ^1^Department of Gynecology, Gynecologic Endocrinology and Oncology, University Hospital of Gießen and Marburg, 35043 Marburg, Germany; ^2^Department of Pathology, University Hospital of Gießen and Marburg, 35043 Marburg, Germany

## Abstract

*Objective*. Vulvar cancer is a rare disease mainly in older women. HPV and non-HPV induced vulvar cancer reflect two types of oncogenesis. Controversies exist on most recent developments in vulvar cancer incidence, patients, and disease characteristics. Changes in incidence, age of disease onset, and tumor site in women treated for primary vulvar cancer in a single German university hospital unit will be described. *Methods*. A retrospective analysis of patient records of women treated between 1994 and 2008 was performed. The fifteen-year-spanning period was divided into three five year-spanning cohorts. Descriptive and statistical analyses were performed. *Results*. 104 patients were identified: cohort-1 from 1994 to 1998 (11 patients); cohort-2 from 1999 to 2003 (21 patients); cohort-3 from 2004 to 2008 (72 patients). Mean age (years) was 73.18 (confidence interval (CI): 64.04; 82.33), 58.9 (CI: 52.24; 65.57), and 61.19 (CI: 57.27; 65.12), respectively. Vulvar cancer confined to the region between clitoris and urethra was seen more often in cohort-3 (*n* = 20) compared to cohort-1 (*n* = 0) or cohort-2 (*n* = 1). *Conclusion*. This analysis supports the notion of rising incidence of vulvar cancer and a changing pattern of anatomical local extension. Disease onset is not restricted to older women.

## 1. Introduction

The incidence of vulvar cancer in women is increasing, especially in younger women [1–4]. The incidence of invasive vulvar cancer is approximately 2.5 per 100 000 women per year in Germany [2]. For the UK similar age-adjusted incidence rates have been reported [5]. In younger women, HPV-infection (mainly type 16) is an important etiological factor for the induction of squamous cell vulvar cancer [6]. Vulvar cancer of the elderly is rarely associated with HPV infection [7], but more often with a history of lichen sclerosus and preneoplastic lesions [4]. The increase of preneoplastic lesions has been reported for some countries [4]. Attention to a woman's report of vulvar symptoms and careful clinical examination of the appropriate anatomical region eventually leading to diagnostic biopsy may contribute to an early detection of preneoplastic lesions or vulvar cancer [8]. Nevertheless, gynecologists are familiar with advanced vulvar cancer requiring complex surgical interventions [9]. The overall survival after 5 years ranges from 87% in stage I to 22% in stage IV [10, 11]. The phenomenon of contralaterally settled metastases originating from one labial site or widely outspread cancer lesions is well known, whereas the occurrence of a vulvar cancer lesion confined to the region between clitoris and urethra is a striking and increasingly seen phenomenon [3]. These observations on vulvar cancer incidence and localisation do not apply for all recent reports [12–15]. The differences described in the aforementioned reports are of highest relevance to the current situation. Thus, further investigations are required to characterise the developments in vulvar cancer which may lead to conclusions for vulvar cancer prevention and early detection.

Here we report changes in incidence and characteristics of vulvar cancer in women treated between 1994 and 2008 in a single German university hospital unit. The potential implications for prevention, screening, and early detection are discussed.

## 2. Material and Methods

A total of 104 women treated for primary vulvar cancer at the Department of Gynecology, Gynecologic Endocrinology and Oncology of the University Hospital Marburg, Germany, between January 1994 and November 2008 were identified. Retrospective analyses were performed; the fifteen-year period was divided into three five-year-spanning intervals to define three cohorts for further analysis. Among others, following parameters were derived from conventional or electronic patient records: age, date of disease onset, histology, tumor stage (FIGO), tumor localisation, previous nonmalignant vulvar lesions, smoking, menopausal status, and surgical procedure. 

Tumor localisation pattern was analysed with respect to anatomical site and number of affected sites. Descriptive analyses of the different parameters with ordinal or nominal scale were performed, showing absolute or relative frequency distribution. Where indicated, mean and standard deviations are shown. 

Age was tested for normal distribution; the differences between the cohorts were tested by ANOVA and post-hoc comparisons with Games-Howell for significance. Kruskal-Wallis-H test and Fisher's exact test were used where indicated.

Statistical evaluations were performed using SPSS for Windows (SPSS Inc. Chicago, IL, USA).

## 3. Results

**Figure 1:**
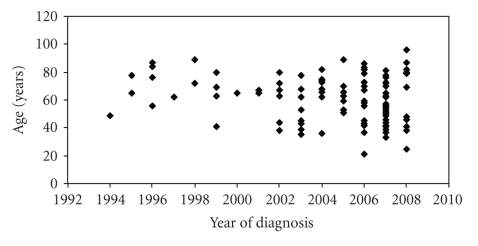
Scatterplot of patient's age at disease onset. The distribution of 104 patients is shown.

**Figure 2:**
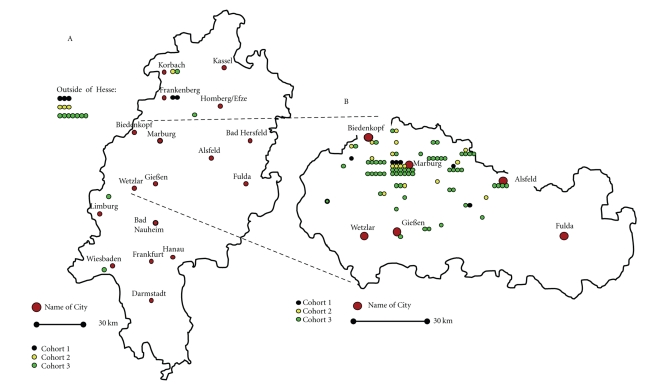
A**:** Residential distribution of vulvar cancer patients treated in Marburg. Map depicts the state of Hesse. B**:** Residential distribution of vulvar cancer patients treated in Marburg. Map depicts the middle of the state of Hesse.

A total of 104 Caucasian women were identified who were treated for primary vulvar cancer at the Department of Gynecology, Gynecologic Endocrinology and Oncology of the University Hospital Marburg, Germany, between January 1994 and November 2008. The number of patients treated since 2005 was maintained on a higher level compared to the numbers in the late nineties of the former century (Figure 1). The regional origin of patients is shown in Figure 2. The regional expansion of patient recruitment did not change during the observation period. The vast majority of patients were derived from counties close to the hospital located in Marburg, Hesse, Germany. The incidence of vulvar cancer increased by six-fold in the county of Marburg-Biedenkopf, raising from approximately 0.7 per 100 000 women per year (calculated from cohort-1) to 4.2 per 100 000 women per year (calculated from cohort-3). The mean age decreased (Table 1) when comparing cohort-3 (year of diagnosis: 2004–2008) with cohort-1 (year of diagnosis: 1994–1998) the difference is significant (*P* = .047; ANOVA) as is between cohort-1 and -2 (*P* = .031; ANOVA). Age distribution is depicted by scatterplot (Figure 1), the median age was 76.0, 63.0, and 62.5 years for cohort-1, -2, and -3, respectively. Cohort-3 included 2 patients younger than 30 years of age. The number of patients younger than 50 years of age increased from *n* = 1 in cohort-1 to *n* = 7 in cohort-2 and *n* = 20 in cohort-3 (*P* = .048; Kruskal-Wallis test).

Among other parameters, tumor stage (FIGO) is shown in Table 1. Tumor depths of infiltration were not different (Table 1). Preneoplastic lesions surrounding the invasive vulvar cancer tissue were detected in 40 (38.5%) of all patients (Table 1). When comparing all women who aged 50 years and older with women younger than 50 years irrespective of the cohort affiliation, preneoplastic lesions surrounding vulvar cancer were found more often in women of <50 years (46.5% (*n* = 13) versus 35.5% (*n* = 27)) although this difference is not significant (*P* = .37; Fisher's exact test).

The affected site distribution pattern was evaluated. The proportion of patients with multiple (two or more anatomical regions) affected sites was lower in cohort 3 (*n* = 37 (51.4%)) compared to cohort-1 (*n* = 10 (88%); *P* = .02, Fisher's exact test) or -2 (*n* = 14(66.6%);0.32, Fisher's exact test).This proportional decrease of multifocal lesions is a consequence of the increase of unifocal lesions as a percentage. The increase of number of patients with vulvar cancer confined to the region between clitoris and urethra is evident, the number is higher in cohort-3 (*n* = 20) compared to cohort-2 (*n* = 1) and cohort-1 (*n* = 0). The differences regarding the number of patients with vulvar cancer confined to the region between clitoris and urethra between cohort-3 and cohort-1 (*P* = .02) or cohort-2 (*P* = .04) were significant (Fisher's exact test). When comparing all women younger than 50 years of age with women equal or older than 50 years irrespective of the cohort affiliation, the vulvar cancer localisation confined to the region between urethra and clitoris revealed a higher proportion in the younger women (*n* = 9 (32.1%) versus *n* = 12 (15.8%); *P* = .06, Fisher's exact test).

Absence or presence of high-risk HPV was recorded in 16 patients with positive evidence of HPV infection in five vulvar cancer samples. During that backdated observation period, HPV testing was not routinely done in vulvar cancer.

Sixty two of all patients had a negative history regarding preneoplastic lesions. Previous lichen sclerosus was known in 22 patients, carcinoma-in-situ in 4 patients. The proportion of patients with a positive history of either of these two lesions is shown in Table 1. When comparing all women who aged 50 years and older with the women younger than 50 years irrespective of the cohort affiliation, the proportion of patients in the group of younger women with a positive history of vulvar lesions was only 3.6% (*n* = 1) compared with the women of the older group (35.5% (*n* = 27)) (*P* = .001; Fisher's exact test).

## 4. Discussion

This retrospective, single centre analysis contributes more data to a most recent relevant discussion on changes in incidence and characteristics of vulvar cancer in women. This single centre 15-year spanning analysis shows an increase of the incidence of vulvar cancer, reveals a decreased median age of the patients at disease onset, and demonstrates an altered disease site distribution pattern. Our data strongly support the report by Hampl et al. [3] with respect to incidence, age of onset and localisation of vulvar cancer.

In the absence of HPV infection, vulvar cancer can emerge from lesions like lichen sclerosus or carcinoma-in-situ [6, 8]. The mutation of tumor suppressor genes like p53 and CDKN2A was found more often in lichen sclerosus derived malignancies than in HPV-induced external genital cancers [16]. In addition to genetic alterations, deterioration in inflammatory response reactions and angiogenesis may develop from lichen sclerosus lesions [17]. An analysis of invasive squamous-cell carcinomas of the vulva demonstrated adjacent lichen sclerosus lesions in 32% of the samples, and with respect to former nomenclature, 77% of the vulvar cancer samples had adjacent VIN lesions [18]. The rate of adjacent VIN lesions was 40.4% in the herein presented collective. Furthermore, 22% of the patients had a positive history for vulvar lichen sclerosus. 

HPV infection is a known predisposing factor leading to vulvar cancer [6, 19]. HPV 16 prevalence was 29.3% in vulvar cancer [20]. Since HPV testing was not routinely performed, one third of the tested samples of vulvar cancer were found positive for high-risk HPV DNA in this series. 

The incidence of vulvar cancer was 1.7 per 100 000 women per year in the USA between 1998 and 2003 [21]. This incidence is slightly lower than reported for Germany [2]. The incidence was increasing over the investigation period [15, 22]. The proportion of women with vulvar cancer younger than 50 years was nearly 20% [12]. In the current cohort, this proportion was 26%. More importantly, the proportion of younger women increased when comparing the diagnosis date-related cohorts in the most recent cohort significantly compared to cohort 1. This observation is in accordance with the data reported by Hampl et al. [3]. This proportional increase of vulvar cancer in women younger than 50 years was not found in the US population-based cancer registries [13–15], although an age-adjusted increase of vulvar cancer incidence over all age groups was reported [23]. The presented data support the notion of an increase in vulvar cancer. The service area of the analyzing hospital did not alter. The number of inhabitants remained fairly constant during the observation period [24] in the county of Marburg-Biedenkopf. The incidence of vulvar cancer increased by six-fold in the county of Marburg-Biedenkopf, raising from approximately 0.7 per 100 000 women per year to 4.2 per 100 000 women per year. Small-area analyses are suitable to detect risk areas [25, 26]. Small area analysis should include a longer observation period thus avoiding misleading results [27]. The observation period of our retrospective analysis is spanning a 15-year interval. The US and the German reports agree in a significant proportion of women younger than 50 years of age out of all vulvar cancer patients. 

Vulvar cancer confined to the urethra-clitoris region was found more often in cohort 3 whereas rarely more than 10 years ago. Similarly, this observation of changes in the tumor localization pattern was reported by Hampl et al. [3]. Vulvar cancer located at the labia majora or minora or at overlapping and multiple sites is the classical presentation of this disease [28, 29]. Until now there is no explanation for this observation [3]. Infection by HPV, chronic or recurrent mechanical irritation, personal life style including sexual behavior and hygiene, cosmetic products, other altered molecular cell survival-regulating pathways leading to initiation and progression of vulvar cancer are among the hypotheses [22, 30, 31]. Increase and change in age distribution of vulvar cancer may require consequences for prevention, screening and detection of early disease. 

Until now, there is no established screening procedure aiming at vulvar cancer, neither for detection of HPV nor of preneoplastic lesions [1]. Regular gynecological examination at least once a year or more often if indicated should contain anamnesis including vulvar symptoms, vulvar inspection, clinical investigations including for inguinal lymph nodes, and in case of suspect lesions performing a vulvoscopy and biopsy to obtain a histological diagnosis [1, 8]. Any persisting or suspect lesion should be clarified by histological investigation, a pap smear is not sufficient [1]. Routine testing for HPV infection in asymptomatic women is not indicated [1]. The surveillance intervals should be scheduled closer in immunocompromised women. Educating women in gynecological diseases is a pivotal measure in cancer prevention and detection [8]. The role of immunotherapies and anti-HPV immunization in the prevention of vulvar cancer is far from being elucidated [1, 10]. 

## 5. Conclusion

Our data confirm an increasing incidence of vulvar cancer and some characteristics of vulvar cancer including age of disease onset and lesion localisation, especially monocentric lesions confined to the clitoris-urethra region. Currently no specific screening procedures or prevention measures can be recommended. Informative education, careful routine gynecological examination at regular intervals and if indicated additional investigations like vulvoscopy and biopsy can contribute to the detection of preneoplastic or early vulvar cancer lesion. Research has to further elucidate the different pathways leading to vulvar cancer, evaluating the preventive and therapeutic potential of immunomodulating measures like topical therapies or HPV vaccination.

## Figures and Tables

**Table 1:** Data of 104 patients with vulvar cancer treated between 1994 and 2008. Figures give number of patients unless noted otherwise.

	Cohort-1 (1994–1998)	Cohort-2 (1999–2003)	Cohort-3 (2004–2008)	
Number	11	21	72	
Mean age [years]	73.18	58.90	61.19	*P* = .047^a^
95% CI [years]	64.04; 82.33	52.24; 65.57	57.27; 65.12	
Age <50 years	1	7	20	*P* = .048^b^
Comorbidities: no	1	3	13	
Tobacco abuse: yes	2	10	25	n.s.^c^

Histology				
Squamous cell	10	17	63	n.s.^b^
Nonsqamous cell		1	4	
Verrucous	1	1	1	
Adenocarcinoma			2	
Other		2	2	

FIGO				
I	2	4	30	n.s.^b^
II	4	4	14	
III	1	3	5	
IV		3	7	
X	4	7	16	

Grading				
G1	3	5	6	n.s.^b^
G2	8	13	57	
G3			6	
G4			1	
GX		3	1	

Depth [mm] mean +/− SD	5.7 (2.1)	3.7 (4.9)	5.3 (5.4)	n.s.^a^

VIN surrounding invasive cancer [%]	9.1	42.8	41.7	*P* = .03^c*^
				*P* = .05^c**^

History of lichen sclerosus or carcinoma-in-situ [%]	45.5	33.3	20.8	n.s.^c^

Unifocal lesion	1	7	35	*P* = .02^c*^

ASA				n.s.^b^
1	1	1	15	
2	1	11	28	
3	3	7	27	
4	0	1	1	
x	6	1	1	

Surgery				*P* = .015^c*^
Biopsy only		2		*P* = .03^c***^
Wide excision or partial vulvectomy	5	11	59	
Radical vulvectomy	6	8	13	

Adjuvant radiation: yes	1	5	14	n.s.^c^

Alive (May 2009)				
Yes	6	20	72	
No		1		
Not known	5			

CI = confidence interval. ASA = American Society of Anesthesiologists' Physical Status Classification. LNE = inguinal lymphadenectomy. SD = standard deviation. ^a^ ANOVA (post-hoc comparisons with Games-Howell). ^b^ Kruskal-Wallis test. ^c^ Fisher's exact test. * Cohort-3 versus cohort-1. ** Cohort-2 versus cohort-1. *** Cohort-2 versus cohort-3.
